# Modified FMCW Scheme for Improved Ultrasonic Positioning and Ranging of Unmanned Ground Vehicles at Distances < 50 mm

**DOI:** 10.3390/s22249899

**Published:** 2022-12-15

**Authors:** Stefano Laureti, Marco Mercuri, David A. Hutchins, Felice Crupi, Marco Ricci

**Affiliations:** 1 Department of Informatics, Modelling, Electronics and Systems Engineering, University of Calabria, Via Pietro Bucci, Arcavacata, 87036 Rende, CS, Italy; 2 School of Engineering, University of Warwick, Coventry CV4 7AL, UK

**Keywords:** ultrasounds, FMCW, correlation, unmanned ground vehicles, ranging, positioning

## Abstract

Unmanned ground vehicles (UGVs) find extensive use in various applications, including that within industrial environments. Efforts have been made to develop cheap, portable, and light-ranging/positioning systems to accurately locate their absolute/relative position and to automatically avoid potential obstacles and/or collisions with other drones. To this aim, a promising solution is the use of ultrasonic systems, which can be set up on UGVs and can potentially output a precise reconstruction of the drone’s surroundings. In this framework, a so-called frequency-modulated continuous wave (FMCW) scheme is widely employed as a distance estimator. However, this technique suffers from low repeatability and accuracy at ranges of less than 50 mm when used in combination with low-resource hardware and commercial narrowband transducers, which is a distance range of the utmost importance to avoid potential collisions and/or imaging UGV surroundings. We hereby propose a modified FMCW-based scheme using an ad hoc time-shift of the reference signal. This was shown to improve performance at ranges below 50 mm while leaving the signal unaltered at greater distances. The capabilities of the modified FMCW were evaluated numerically and experimentally. A dramatic enhancement in performance was found for the proposed FMCW with respect to its standard counterpart, which is very close to that of the correlation approach. This work paves the way for the future use of FMCWs in applications requiring high precision.

## 1. Introduction

As the use of unmanned ground vehicles (UGVs) and unmanned aerial vehicles (UAVs, also known as “drones”) is currently increasing, extended efforts have been made to develop systems for tracking and determining their position, both outdoors and indoors. While multiple UGVs and drones can be tracked and located outdoors using global navigation satellite system (GNSS) receivers, the accuracy provided is often insufficient at distances below 1000 mm, and this is dramatically reduced in indoor environments due to the lack of satellite coverage [1].

Existing measurement technologies for indoor tracking and positioning include wireless sensor networks [2,3], radio frequency-based systems [4,5,6,7], video-based localization systems [8,9], infrared positioning systems [10,11,12], wireless local area networks [13,14,15] and Bluetooth-based systems [16,17]. The reader is referred to, e.g., Mainetti et al. [18] or to Koyuncu et al. [19], for a thorough comparison of such measurement approaches in terms of hardware requirements, cost, working range, measurement resolution, and repeatability.

In addition, ultrasonic transmitters (Tx) and receivers (Rx) can be fixed to both UGVs and UAVs as a reliable, light, and inexpensive solution. They can be used to accurately estimate the distance from/to a specific cartesian origin to track their relative positions, and to map the surrounding environment [20,21]. It is worth noting that UGVs are often used within industrial environments; therefore, the use of ultrasound for range and positioning applications instead of electromagnetic methods is beneficial for avoiding unwanted interaction with other electromagnetic sources or metal parts and structures [22], especially when these are aimed at being used for application with high accuracy. 

The basis of an ultrasonic ranging system is the accurate and rapid estimation of the time-of-flight (TOF), which is the time taken for the ultrasonic stimulus to travel from the Tx to the Rx. The distance between the Tx and Rx, i.e., the distance either between drones or from a drone to a given target/obstacle, is then inferred from the TOF using the speed of sound in air, which is 343 m/s at room temperature [23]. However, an accurate estimation of the TOF relies on the use of advanced signal conditioning and post-processing algorithms. In such cases, the use of frequency-modulated swept-sine “chirp” signals as the excitation waveform has been shown to be of great help for TOF estimation, especially in poor signal-to-noise ratio (SNR) conditions [24]. However, this is difficult to use when inexpensive narrowband ultrasonic transducers and low-cost hardware are employed.

Different post-processing strategies have been developed to further improve the range estimation capabilities when using such coded signals, among which the correlation method, also known as pulse-compression (PuC) [25,26], and the frequency-modulated continuous waveform (FMCW) [27,28,29] are the most frequently employed. The former provides the highest accuracy and repeatability of TOF estimation, although it is based on a convolution procedure and, thus, requires relatively powerful hardware to be correctly performed. The latter relaxes several hardware constraints at the cost of reduced accuracy and repeatability, especially when using narrowband transducers and low-cost hardware.

In general, correlation-based estimation is the best choice for ranging and positioning applications [30]. However, when cheap, light, portable, and low-power ultrasonic ranging and positioning systems are aimed at being set up, e.g., on drones, FMCWs can be a trade-off solution between the design constraints and the TOF estimation accuracy and repeatability.

Several studies have focused on reducing the computational requirements of the correlation, to explore alternative post-processing strategies, and to compare the capabilities of the correlation and the FMCW methods. Barshan [31] compared four different methods for distance estimation in the range of 100–5000 mm, i.e., simple thresholding, curve-fitting, sliding-window, and correlation, and found that, whereas the correlation is a theoretical optimal in terms of bias error, standard deviation, total error, and robustness to noise, the other algorithms offer enough performances at a much lower computational cost. Huang et al. [32] used binary frequency shift-keyed signals as excitation waveforms plus phase detection to improve TOF estimation in the range of 1000–6000 mm and found a range accuracy within ± 0.05 mm. Hazas and Hopper [33] prototyped a broadband “Dolphin” transmitter and receiver unit to overcome the limitation of using narrowband ultrasonic transducers in the range of 500–2500 mm. Saad et al. [34] explored the use of wideband frequency-hopping spread spectrum techniques in combination with a minimum variance search technique to correct the error in correlation-based TOF measurement, yielding an improvement in the estimations within the range 500–7000 mm. Jackson et al. [35] compared several time-domain approaches with their frequency-domain counterparts, focusing especially on the accuracy and repeatability of the TOF measurements. Additionally, the use of a hybrid time-frequency domain and biologically inspired models was investigated for measurement in the range of 100–1000 mm. Ronkin et al. [36] introduced a novel approach for FMCW-based TOF estimators relying on a heterodyne scheme, which showed improved performances over the above-reported processing algorithms. Efforts have also been made to make ultrasonic FMCW-based systems highly portable, light, and based on low-power hardware (see, for instance, Berkol et al. [37] or De Angelis et al. [38]) and to combine the ultrasound ranging with infrared technology to improve the overall accuracy [39] in the range of 50–1000 mm and 500–1400 mm, respectively.

It can be noticed from the literature that few authors have used ultrasonic FMCWs for very small *TOF* estimation at distances below 50 mm in air. In fact, FMCW suffers from very low accuracy and repeatability when the Tx and Rx are very close together with respect to correlation-based estimation, especially when a relatively-low sampling frequency and narrowband transducers are employed [40]. The TOF resolution lower bound for the FMCW technique is 1/B, with B being the transducer bandwidth [28,30]. However, being able to effectively exploit the ultrasonic FMCW technique, even for very close targets, would be of interest for the tracking and positioning of UGVs and drones within a fleet to avoid potential collisions and to faithfully map their surrounding environment. It is worth mentioning that ultrasound is widely used in a plethora of different applications, such as nondestructive testing and evaluation [41,42,43], sonochemistry [44], medical treatment [45,46,47,48], and imaging [49], so several ultrasonic ranges and positioning schemes benefit from research in these areas.

This paper introduces a modified FMCW measurement scheme that dramatically improves TOF estimation for distances < 50 mm, i.e., below the theoretical limit of the 1/B of the current setup, which can be implemented via simple and portable signal generation and acquisition hardware.

The capabilities of such an approach will be evaluated numerically and experimentally considering both the features of suitable hardware and the central frequency/bandwidth of widely-available ultrasonic sensors, such as the one commonly employed in parking sensors. By establishing ad hoc figures of merit, the standard and modified FMCW-based approaches are compared to the correlation-based algorithm in terms of accuracy and repeatability for several different distances in air and different SNR levels so as to mimic different attenuation levels. Additionally, a three-point parabolic fit [30] will be implemented on the estimated TOFs to simulate the results of using a phase-locked loop (PLL) in a frequency-demodulation configuration for a precise reading of the frequency beat value [50].

The paper is organized as follows. Section 2 details the features of the simulated hardware and transducers; the theory and working principle of the correlation and standard FMCW methods are discussed in Section 3, together with the modified FMCW scheme proposed here; Section 4 explains the numerical simulation procedure in detail and contains a thorough description of the figures of merit. The numerical and experimental results are shown and discussed in Section 5. Section 6 draws up conclusions and addresses potential future work.

## 2. Detail of the Simulated Hardware and Setup

A plethora of signal generators, digital-to-analog (DACs) and analog-to-digital (ADCs) converters, and transducers are available in the market to set up ultrasonic ranging and positioning systems. However, the choice of the equipment specified below is thought to be suitable for enabling the proposed FMCW measurement scheme to be implemented using inexpensive and lightweight hardware. In fact, while the former aspect is, in general, related to the available budget, the choice of lightweight equipment is beneficial to fully exploit the potential of a given UAV. In order to gain more insight, Table 1 shows an accepted taxonomy of UAVs [51]. It can be noticed that micro, mini, and even small UAVs will benefit from the minimization of hardware weight, as both their endurance and payload can be maintained close to the nominal values. Note that the same rationale holds for UGVs.

To this aim, the specifics of a Digilent Analog Discovery 2 (Digilent—National Instrument, Austin, TX, USA) have been simulated here, and the same hardware was used for the experimental activity. This device has a nominal weight of 450 g and a cost of less than USD 400. It provides two DAC arbitrary waveform generators (AWGs) that can be driven at a maximum output voltage of 10 V peak-to-peak, two ADCs channels with 14 bits of resolution, and a maximum buffer of 8000 samples for generating/acquiring the signals at the sampling frequencies $f_{ADC}$ and $f_{DAC}$, respectively, at up to 100 MSamples/s. Note that the chosen $f_{ADC}$ and $f_{DAC}$ values are much lower than the maximum value, i.e., 500 kSamples/s, so as to allow more inexpensive hardware to be chosen if required in the future.

Concerning the ultrasonic transducers, a pair of Murata MA40S4S transducers (Murata Manufacturing Co., Ltd., Kyoto, Japan) were chosen for study. These are optimized for indoor sensing purposes and have a 7.1 mm diameter active element size, a resonance central frequency $f_{c}$ equal to 40 kHz, and a −3 dB bandwidth B equal to 5 kHz, all for a cost of less than USD 6 and a weight of about 30 g each.

**Figure 1:**
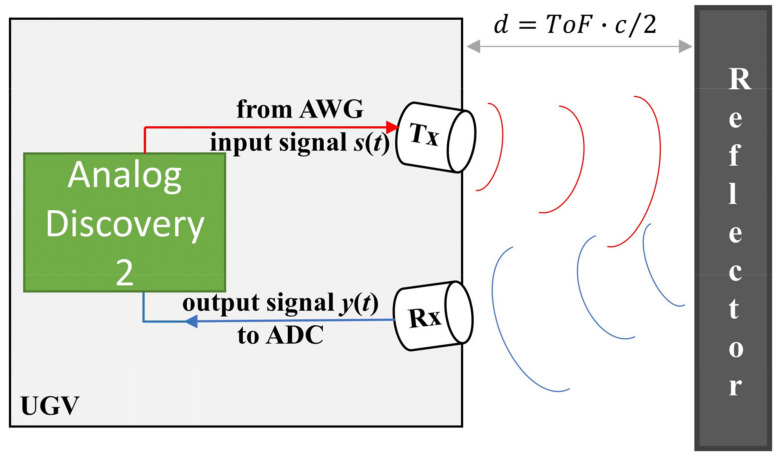
A sketch of the simulated ultrasonic ranging and positioning system.

A sketch of the possible implementation of this hardware onto the top/bottom surface of a UGV is depicted in Figure 1.

Figure 1 shows that measuring the distance d between a potential sound reflector close to the UGV, e.g., other UGVs or an obstacle, relies on the accurate estimation of TOF from the output signal y(t), with TOF being the time taken by the input ultrasonic signal s(t) to travel from the Tx to the Rx after reflection from an object. Once the TOF is estimated, the distance d is obtained using Equation (1).

(1) $$d=\frac{TOF\cdot c}{2}.$$

Note that a change in air temperature would result in a change in the speed of sound, c, hence influencing the correct estimation of the distance. However, this detrimental effect can be considered negligible when relatively-low TOF values are being estimated, e.g., for d < 50 mm, as in the present case. 

Different excitation signals, s(t), can be practically employed for estimating TOF, such as simple pulsed excitation, pseudo-noise and chirp signals, stepped-frequency continuous wave signals, etc. However, chirp signals are very often used in ultrasonic positioning and ranging applications for their design flexibility and beneficial features. The next sections will highlight the benefit of such an approach with respect to a simple pulsed excitation, especially in correlation- and FMCW-based schemes in combination with relatively low peak-to-peak input voltages.

## 3. Theoretical Background

### 3.1. Linear Swept-Frequency Chirp Signal

The use of frequency-modulated coded signals is well-established in several ultrasonic applications, including ultrasonic-based range and positioning systems [52]. In fact, chirp signals can be easily designed so as to cope with the useful transducer’s bandwidth for an arbitrary duration, and they inherently establish a relationship between time and frequency, thus giving a unique advantage in both correlation- and FMCW-based estimators. On top of these, the chirp autocorrelation function is *δ*-like, which is of the utmost importance in correlation-PuC measurement schemes, as explained in detail in the next subsections.

The general formulation of a chirp signal, s(t), is reported in Equation (2).

(2) $$s\left(t\right)=Re\left[e^{2\pi i\left(f_{c}t+\frac{B}{2}\int_{0}^{t}x\left(\tau \right)d\tau \right)}\right]=Re\left[e^{i\Phi \left(t\right)}\right],$$

with Φ(t) being a nonlinear phase function of the time, x(τ) is a monotonically increasing, smooth modulating signal taking values in [−1,1], t∈[0,T] so that T is the chirp time duration, $f_{c}=\frac{f_{1}+f_{2}}{2}$ is the central frequency of the chirp, and $B=f_{2}-f_{1}$ is the bandwidth, with $f_{1}$ and $f_{2}$ being the start and stop frequency of the chirp, respectively; Re stands for the real part operator. The instantaneous frequency of s(t) is defined as $f_{inst}\left(t\right)=\frac{1}{2\pi }\frac{d\Phi \left(t\right)}{dt}$. If the phase Φ(t) is chosen so as to be a quadratic function of the time, as per Equation (3) [42], then:

(3) $$\Phi \left(t\right)=2\pi \left(f_{c}t+\frac{B}{2T}t^{2}-\frac{B}{2}t\right)=2\pi \left(f_{1}t+\frac{B}{2T}t^{2}\right),$$

then, a linear chirp is obtained:

(4) $$s\left(t\right)=Re\left[e^{2\pi i\left(f_{1}t+\frac{B}{2T}\;t^{2}\right)}\right],$$

whose $f_{inst}\left(t\right)$ in t∈[0,T] is:

(5) $$f_{inst}\left(t\right)=f_{1}+\frac{B}{T}t=f_{1}+k_{f}t,$$

with $k_{f}=\frac{B}{T}$.

**Figure 2:**
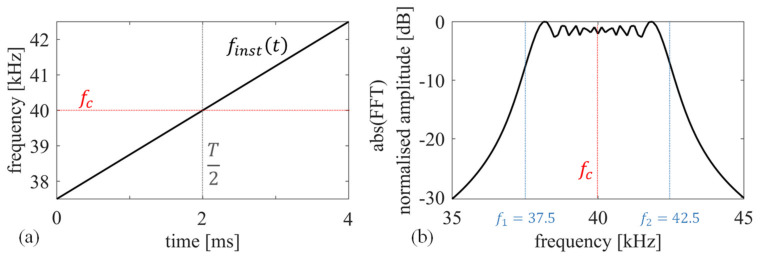
(**a**) Time-frequency handling for a chirp signal, sweeping from 37.5 kHz to 42.5 kHz in 4 ms, and (**b**) its frequency spectrum.

Figure 2a shows an example of the $f_{inst}\left(t\right)$ for a linear chirp signal with $f_{1}\;$= 47.5 kHz, $f_{2}\;$= 52.5 kHz, and T = 4 ms, while Figure 2b depicts its frequency spectrum obtained using the fast Fourier transform (FFT) algorithm.

Note that these chirp characteristics are used throughout the manuscript, as they align with the chosen transducer’s nominal bandwidth.

### 3.2. Pulse-Compression/Correlation Basic Theory

PuC is a measurement technique that is largely employed to experimentally estimate the impulse response, h(t), of a linear time-invariant (LTI) system, which is highly beneficial when dealing with poor SNR values.

PuC uses a coded excitation, s(t), and another signal, Ψ(t), the so-called matched-filter, such that their convolution (∗) approximates the Dirac’s delta function, δ(t), so that $[s*\Psi ]\;\left(t\right)=\tilde{\delta }\left(t\right)\sim \delta \left(t\right)$. The impulse response, h(t), is thus estimated by exciting the LTI system with the signal s(t) and then convolving the system output y(t) with Ψ(t). Note that the linear chirp signal described in Section 3.1 is largely employed in PuC-based measurements, as its autocorrelation function is $\tilde{\delta }\left(t\right)$.

PuC can be mathematically demonstrated considering an output signal y(t), physically resulting from the convolution between the input signal s(t) and the system’s impulse response h(t), on which is superimposed an additive white Gaussian noise (AWGN) e(t): y(t)=[h∗s](t)+e(t), with Ψ(t) and e(t) assumed to be uncorrelated. In practical terms, s(t) is the chirp signal generated from the Tx, whilst y(t) is just the signal acquired from the Rx ultrasonic transducer and is digitized via the ADC.

An estimate, $\tilde{h}\left(t\right)$, of the impulse response h(t) is then obtained, convolving y(t) with the matched filter, Ψ(t):

(6) $$\tilde{h}\left(t\right)=\left[y*\Psi \right]\left(t\right)=h\left(t\right)*\underset{\sim \delta \left(t\right)}{[\underset{︸}{s*\Psi ]\left(t\right)}}+\left[e*\Psi \right]\left(t\right)\sim \left[h*\delta \right]\left(t\right)+\tilde{e}\left(t\right)=h\left(t\right)+\tilde{e}\left(t\right),$$

with $\tilde{e}\left(t\right)$ also being AWGN but with lower energy than e(t), hence resulting in an increased SNR level. Note that, in the case of a periodic excitation, Equation (6) is a *cyclic* correlation and can be easily implemented in the frequency domain as per Equation (7):

(7) $$\tilde{h}\left[n\right]=DFT^{-1}\left\{DFT\left\{y\left[n\right]\right\}\times {\left(DFT\left\{s\left[n\right]\right\}\right)}^{f}\right\},$$

where digital signals have also been considered, hence DFT stands for discrete fourier transform, and “f” for complex-conjugate. The reader is referred to Hutchins et al. [53] or to Misaridis et al. [54] for a thorough description of the PuC algorithm in ultrasonic applications. The main advantage of using the PuC scheme in combination with chirp signals is that the SNR of the estimated $\tilde{h}\left(t\right)$ can be increased with respect to, e.g., the easier use of a standard pulsed excitation while assuring the same or even better range resolution. This beneficial effect happens when the energy of the chirp signal is larger than that of a standard pulsed excitation and the same, or a larger bandwidth is excited by the chirp. It should be noted that hardware constraints play a key role here: the maximum time duration of both the chirp $T_{chirp}$ and of a pulse signal $T_{pulse}$ are constrained by the available buffer size and the $f_{DAC}$ of Analog Discovery 2, i.e., T, and so is the maximum amplitude (10 V peak-to-peak). On top of that, the available B is dictated by the ultrasonic transducers, in this case, 5 kHz. However, for a standard pulse of duration $T_{pulse}$, the excited bandwidth is $B\propto \frac{1}{T_{pulse}}$, so that $T_{pulse}$ is very unlikely to be equal in value to T. The same does not hold for the chirp signal, as the duration, $T_{chirp},$ is not directly constrained by B, and its duration can be as long as the available T of the hardware, see Equation (5). If the pulse amplitude is constant and equal to $A_{pulse}$, the pulse energy is $E_{pulse}=A_{pulse}T_{pulse}=\frac{A_{pulse}}{B}$ while the chirp energy is $E_{Chirp}=A_{Chirp}T_{Chirp}$. Choosing $A_{pulse}=A_{Chirp}=$ 10 V, the maximum available SNR gain is

(8) $$SNR_{gain}=\frac{SNR_{PuC-Chirp}}{SNR_{pulse}}=\frac{E_{chirp}}{E_{pulse}}=\frac{A_{Chirp}}{A_{pulse}}\cdot T\cdot B=T\cdot B$$

Thus, the available SNR gain is proportional to the time-bandwidth product (T·B) of the coded signal [55], hence the use of a chirp signal instead of a simple pulse. 

It has been found that, in terms of SNR, the best choice for the matched filter is simply Ψ(t)=s(−t) [56], i.e., employing the time reversal of the input signal as the matched filter. 

**Figure 3:**
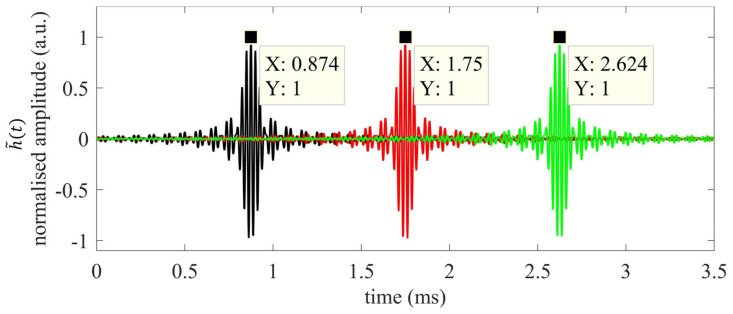
Simulated crosscorrelation outputs for reflectors at d1= 300 mm (black), d2= 600 mm (red), and d3= 900 mm (green) from the transducer pair.

Thus, Equations (6) and (7) are simply a correlation between the received signal and the matched filter, where the maximum amplitude of the correlation output is at t=0 if no delay between the two signals is found, *i.e.,* ToF=0 s. When a reflector is placed at d>0 mm, then the correlation peak of $\tilde{h}\left(t\right)$ obtained from Equation (6) shifts accordingly. This concept is shown in Figure 3, where three waveforms (y(t)) have been time-shifted so as to mimic the reflectors at distances equal to d1= 300 mm, d2= 600 mm, and d3= 900 mm, using the same chirp parameters as in Figure 2. Note that for the sake of clarity, no sound attenuation is here taken into account. The different ToFs are, thus, estimated via the correlation-based approach as per Equation (9):

(9) $$TOF=\underset{t}{\mathrm{argmax}}\;\left[\tilde{h}\left(t\right)\right],$$

thus, obtaining $ToF_{d1}^{Corr}$, $ToF_{d2}^{Corr}$, $ToF_{d3}^{FMCW}$ of 0.874 ms, 1.750 ms, and 2.624 ms, respectively. By substituting the estimated TOFs into Equation (1), distances of 299.78 mm, 600.25 mm, and 900.03 mm are obtained.

It is worth noting that the estimated distances are slightly different from the simulated ones, and this error becomes larger for relatively low distances. Nevertheless, the error can be reduced by using several post-processing techniques, such as interpolation, so as to better estimate the sample/time position of the correlation peak. These can be applied both for correlation- and FMCW-based estimation. In this paper, only a parabolic interpolation procedure will be exploited—the idea here is to propose a workaround based on a very simple approach that can be directly implemented onboard with simple hardware and without using any additional postprocessing. The interested reader is referred to, e.g., Svilainis et al. [30], for details of such post-processing and for a thorough overview of the influence of the hardware parameters on the error. 

### 3.3. FMCW Basic Theory

**Figure 4:**
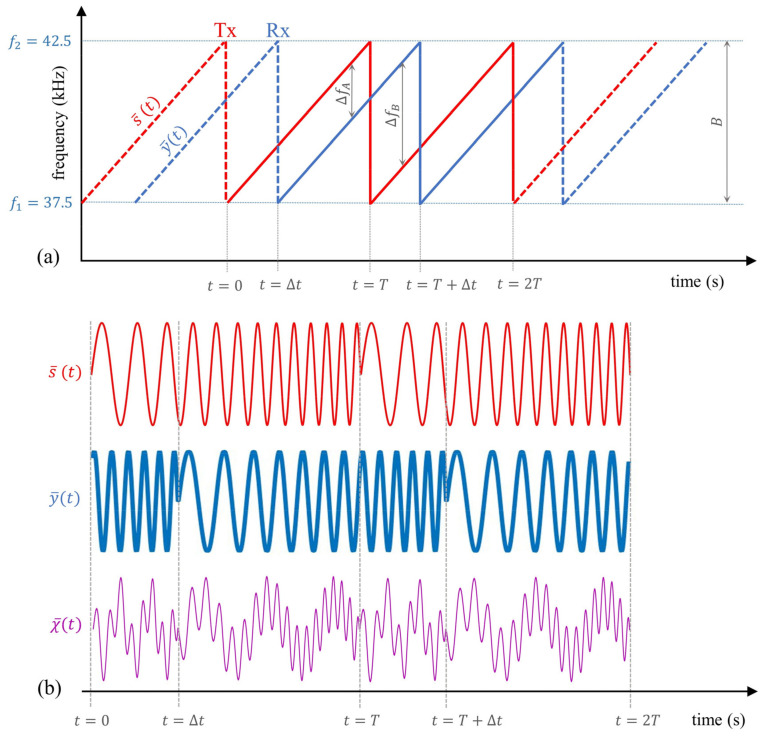
(**a**) $\bar{s}\left(t\right)$ (red) and $\bar{y}\left(t\right)$ (blue) signals’ $f_{inst}\left(t\right)$. Note that $\bar{y}\left(t\right)$ is time-shifted (Δt) replica of the transmitted signal $\bar{s}\left(t\right)$; (**b**) an example of the mixed output signal, $\bar{\chi }\left(t\right)$ (purple).

In FMCW-based estimation, the Tx is excited via a periodic chirp signal—at least two periods are needed—$\bar{s}\left(t\right)$, and the Rx receives the output $\bar{y}\left(t\right)$ after being reflected from a potential reflector. In the ideal noise-free situation, $\bar{y}\left(t\right)$ can be assumed to be just a shifted replica of $\bar{s}\left(t\right)$, $\bar{y}\left(t\right)$= $\bar{s}\left(t-\Delta t\right)$. In order to retrieve the TOF value, $\bar{y}\left(t\right)$ is multiplied by $\bar{s}\left(t\right)$, thus obtaining a periodically mixed signal $\bar{\chi }\left(t\right)$ that contains a beat tone whose frequency is strictly related to the time shift between the Tx and Rx signals. In fact, if Δt > 0, then $\bar{s}\left(t\right)$ and $\bar{y}\left(t\right)$ have different values for instantaneous frequency, $f_{inst}\left(t\right)$, and a beat frequency is produced by mixing them up [57]. In order to gain a better insight into this, Figure 4 shows a sketch of the FMCW basic working principle.

Figure 4a shows that a time delay, Δt, between $\bar{x}\left(t\right)$ and $\bar{y}\left(t\right)$ results in the two values for the instantaneous frequency differences $\Delta f_{A}$ and $\Delta f_{B}$ in a single period T since a fictitious time shift $\bar{\Delta t\;}$= (T−Δt) appears due to the periodic nature of the signals. Thus, $\Delta f_{A}$ and $\Delta f_{B}$ are equal to

(10) $$\Delta f_{A}=k_{f}\;\Delta t,\;\Delta f_{B}=k_{f}\;\bar{\Delta t}=k_{f}\left(T-\Delta t\right)=B-\Delta f_{A}.$$

Hence, the delay Δt can be estimated by measuring $\Delta f_{a}$ and/or $\Delta f_{b}$ through a Fourier analysis applied to $\bar{\chi }\left(t\right)$.

In order to understand how this is possible, the nature of the mixed signal $\bar{\chi }\left(t\right)$ must be analyzed in more detail. By considering Equation (4), and a single chirp period other than the first, the two chirp signals can be written as

(11) $$\bar{s}\left(t\right)=Re\left[e^{2\pi i\left(f_{1}t+\frac{B}{2T}\;t^{2}\right)}\right],\;\mathrm{for}\;t\in \left[0,T\right]\;\bar{y}\left(t\right)=\{\begin{array}{c} \;Re\left[e^{2\pi i\left(f_{1}\left(t+T-\Delta t\right)+\frac{B}{2T}{\left(\;t+T-\Delta t\right)}^{2}\right)}\right],\;\mathrm{for}\;t\in \left[0,\Delta t\right]\\ Re\left[e^{2\pi i\left(f_{1}\left(t-\Delta t\right)+\frac{B}{2T}{\left(\;t-\Delta t\right)}^{2}\right)}\right],\;\mathrm{for}\;t\in \left[\Delta t,T\right] \end{array},$$

so that $\bar{\chi }\left(t\right)$ is:

(12) $$\bar{\chi }\left(t\right)=\bar{y}\left(t\right)\cdot \bar{s}\left(t\right)\;=\{\begin{array}{l} \frac{1}{2}Re\left\{e^{-2\pi i\left[f_{1}\bar{\Delta t}-\frac{B}{2T}\;{\bar{\Delta t}}^{2}\right]}\cdot e^{2\pi i\;\frac{B}{T}\;t\;\bar{\Delta t}}\right\}+\frac{1}{2}Re\left\{e^{4\pi i\left[f_{1}t+\frac{B}{2T}\;t^{2}\right]}\cdot e^{2\pi i\left[-f_{1}\bar{\Delta t}+\frac{B}{2T}\;{\bar{\Delta t}}^{2}\right]}\cdot e^{-2\pi i\;\frac{B}{T}\;t\;\bar{\Delta t}}\right\},\mathrm{for}\;t\in \left[0,\Delta t\right]\\ \frac{1}{2}Re\left\{e^{-2\pi i\left[f_{1}\Delta t-\frac{B}{2T}\;\Delta t^{2}\right]}\cdot e^{2\pi i\;\frac{B}{T}\;t\;\Delta t}\right\}+\frac{1}{2}Re\left\{e^{4\pi i\left[f_{1}t+\frac{B}{2T}\;t^{2}\right]}\cdot e^{2\pi i\left[-f_{1}\Delta t+\frac{B}{2T}\;\Delta t^{2}\right]}\cdot e^{-2\pi i\;\frac{B}{T}\;t\;\Delta t}\right\},\mathrm{for}\;t\in \left[\Delta t,T\right] \end{array}.$$

Equation (12) shows that $\bar{\chi }\left(t\right)$ is the superposition of (i) a sinusoidal low-frequency signal and (ii) that of a chirp signal in both the intervals [0,Δt] and [Δt,T]. For t∈[0,Δt], the frequency of the sinusoidal signal is $\Delta f_{B}$, while for t∈[Δt,T], the frequency of the sinusoidal signal is $\Delta f_{A}$. Thus, the TOF can be retrieved by applying the Fourier analysis on $\bar{\chi }\left(t\right)$ and then measuring the frequency value at which the peak of the sinusoidal low-frequency signal happens, provided that (i) and (ii) are nonoverlapping in their frequency.

Hence, the FMCW measurement protocol consists of (1) mixing the input and output signals $\bar{y}\left(t\right)$ and $\bar{s}\left(t\right)$, (2) filtering the mixer output $\bar{\chi }\left(t\right)$ with a low-pass filter to remove the high-frequency chirp component, (3) converting the resulting signal into a digital one $\bar{\chi }\left[n\right]$ via the ADC, (4) calculating the DFT of $\bar{\chi }\left[n\right],$ and then estimating the frequency value for which the highest peak in frequency is met via

(13) $$\Delta f=\underset{n}{\mathrm{argmax}}\;\left[DFT\left(\bar{\chi }\left[n\right]\right)\right].$$

The TOF is then obtained by substituting Equation (1) into Equation (13):

(14) $$\Delta f=k_{f}\;\Delta t=\frac{B}{T}\;\cdot 2\cdot \;\frac{d}{c}\;\;\;\;⟶\;\;\;\;\;TOF=\frac{T}{B}\Delta f.$$

**Figure 5:**
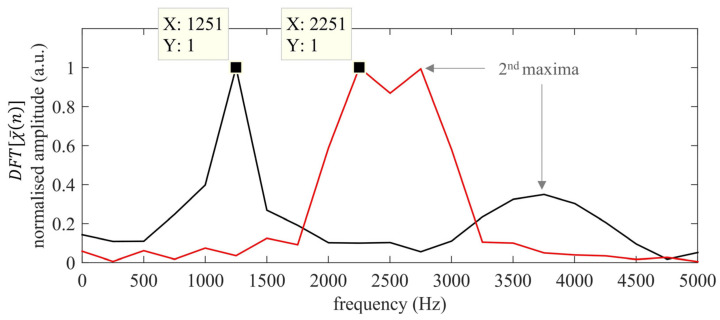
$ABS\left(DFT\left(\bar{\chi }\left[n\right]\right)\right)$ obtained by simulating reflectors at d1= 300 mm (black) and d2= 600 mm (red).

It should be noted that the presence of the two “complementary” beat frequencies for each period leads to an ambiguity in TOF estimation, so that Δf can be either $\Delta f_{A}$ or $\Delta f_{B}$ depending on Δt being smaller or larger than $\frac{T}{2}$. This is because the two different delay values, Δt and $\Delta t^{\prime }=T-\Delta t,$ give the same $DFT\left(\bar{\chi }\left[n\right]\right)$. Thus, T must be chosen so that the maximum expected delay/distance obeys $\Delta t_{max}<\frac{T}{2}$ so that the TOF can be unambiguously retrieved by using Equation (13). Under this constraint, and for a fixed inspection range, the PuC approach guarantees a double repetition rate with respect FMCWs. As an example, Figure 5 shows the $DFT\left(\bar{\chi }\left[n\right]\right)$ obtained for the same distances shown for the PuC case. Note that $\Delta t_{max}<\frac{T}{2}$, hence d3 is out of the current measurement range.

Using Equation (14), a $ToF_{d1}^{FMCW}$ and $ToF_{d2}^{FMCW}$ equal in value to 1.000 ms and 1.800 ms are obtained respectively, which in turn correspond to distances of 343.27 mm and 617.67 mm. A first comparison of the estimated distance values for both the FMCWs and the correlation strategies shows that the latter approach results in better estimates, and this is in line with what has been shown in several research works [52,58]. However, the FMCW-based approach outputs good results, and these can be useful in several different ranges and positioning applications, especially when low hardware resources are available/needed, as is true for the present case. In fact, note that the beating frequencies values can be faithfully captured even with lower $f_{ADC}$ values, thus relaxing (even more) the requested hardware feature.

### 3.4. Limits of the FMCW Approach and the Proposed FMCW-Modified Scheme

The estimated values of $ToF_{d1}^{FMCW}$ and $ToF_{d2}^{FMCW}$ suggest that the lower the real-time TOF to be estimated, the higher the relative error. In fact, for $\Delta t<\frac{1}{B}$, $\Delta f=\frac{B}{T}\Delta t<\frac{1}{T}=f_{REP}$, meaning that the low-frequency interference term does not complete a whole cycle within the excitation period T. This, in turn, hampers the correct measurement of Δf. For the current setup, B is equal to 5 kHz, and thus any Δt value that is less than 0.2 ms is below the FMCW resolution. Note that this value corresponds to a critical distance range of 0–68.6 mm in air.

In order to overcome this limitation, a modified FMCW-based measurement scheme is proposed here. This relies on simply using a shifted replica, $\bar{s}\left(t+t_{0}\right)$, of the input signal, $\bar{s}\left(t\right),$ for the mixing stage. This strategy allows for obtaining a $\Delta f>f_{REP}\;\forall \;\Delta t$ provided that $t_{0}>\frac{1}{B}$, thus assuring at least a whole interference period is to be completed for T. The procedure is reported in Equation (15) for a single excitation period so as to be easily comparable to the standard FMCW approach, as per Equation (12):

(15) $$\bar{\chi }\left(t\right)=\bar{y}\left(t\right)\cdot \bar{s}\left(t+t_{0}\right).$$

**Figure 6:**
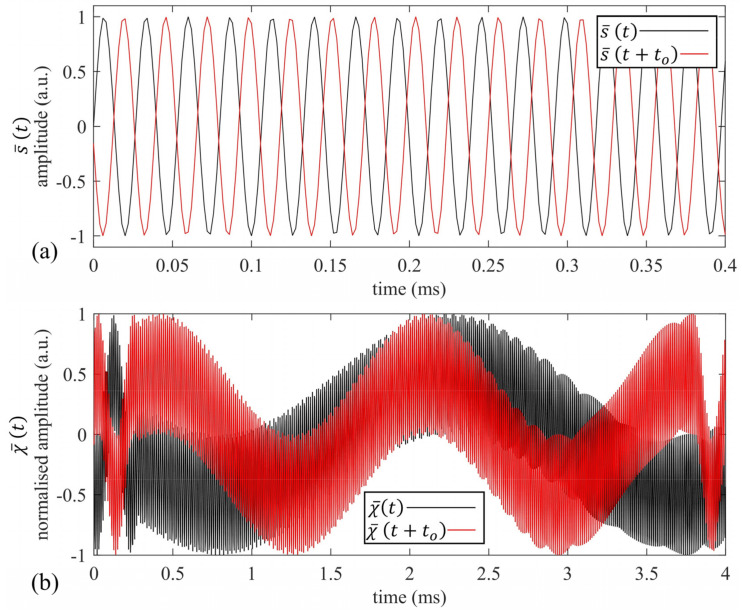
(**a**) A zoom of the $\bar{s}\left(t\right)$ (black) and $\bar{s}\left(t+t_{0}\right)$ signals (red); (**b**) $\bar{\chi }\left(t\right)$ (black) and $\bar{\chi }\left(t+t_{0}\right)$ for the simulated target distance of 50 mm.

In order to gain more insight into the proposed procedure, $\bar{y}\left(t\right)$ is simulated to be the output ultrasonic signal coming from a reflector at a distance d = 50 mm, i.e., within the Δt’s critical range < $\frac{1}{B}$. Figure 6a shows both $\bar{s}\left(t\right)$ and $\bar{s}\left(t+t_{0}\right)$ in the first 0.4 ms of the 4 ms overall duration, while the corresponding $\bar{\chi }\left(t\right)$ and the resultant $DFT\left(\bar{\chi }\left[n\right]\right)$ values are depicted in Figure 6b. Here, $t_{0}$ was chosen to be 0.2 ms, i.e., exactly equal in value to the critical $\frac{1}{B}$ bound. This simple example shows the potential of the proposed approach for the accurate estimation of distances <68.6 mm; the modified approach completes a whole interference cycle within the chirp period (Figure 6b, red plot), while the same is not true for the standard FMCW (Figure 6a, black plot). It is worth noting that this modified scheme can be easily implemented by storing a shifted replica of the input signal in the second available AWG buffer.

## 4. Details of the Numerical Simulations and Figures of Merit

A MATLAB routine has been established to compare the capabilities of the correlation- and the two FMCW-based approaches to estimate the different TOF values and hence the measurement of different distances, d, in air. In particular, two chirp signals, having T = 4 ms, B = 5 kHz, and $f_{c}\;$= 40 kHz, were generated at $f_{DAQ}\;$=$\;f_{ADC}\;$= 500 kHz and sent together so as to have a periodic excitation. Note again that the chosen T value matches the maximum available samples of the Analog Discovery 2 device and that the characteristics of the chirps match those of the chosen transducers. 

In order to consider the constraints of the FMCW-based approaches, a maximum measurable distance range of $\frac{T}{2}\times c≅$ 680 mm has been simulated. The signals $\bar{y}\left[n\right]$ and $\bar{\chi }\left[n\right]$ have been simulated by generating a signal $\bar{s}\left[n\prime \right]\sim \bar{s}\left(t\right)$ at $f_{DAQ}$ and then also generating a $\bar{y}\left[n\prime \right]$ signal with the same rate and with $N_{\Delta t}=201$ different Δt delays linearly-distributed in the interval Δt∈[0,T/2) so as to sweep the range of distances d∈[0,680) mm at increasing increments of 3.4 mm. Note also that a $\bar{s}\left(t+t_{0}\right)$ has been generated for the modified FMCW-based approach in the same way, whereby the nonzero phase shift has been chosen to be $t_{0}=1/B$ = 0.2 ms. 

**Figure 7:**
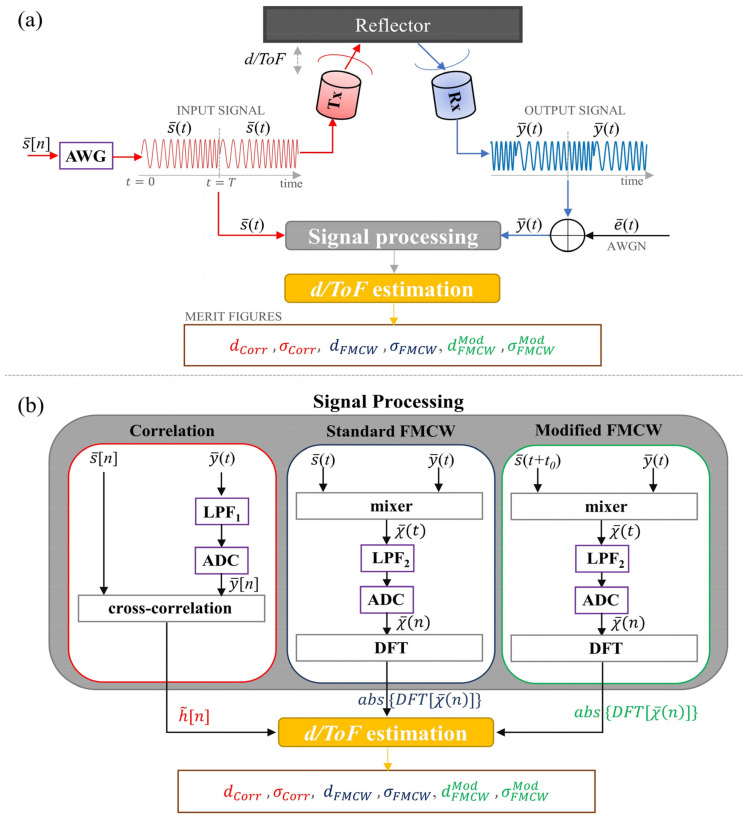
(**a**) Sketch of the implemented routine and retrieved figures of merit; (**b**) details of the implemented postprocessing for each of the three estimation methods.

In order to simulate the effect of noise, $N_{trials}=5000$ AWGN noise patterns were generated at $f_{ADC}$ and added to $\bar{y}\left[n\prime \right]$ to finally obtain $\bar{y}\left[n\prime \right]\sim \bar{y}\left(t\right)\;$ and $\bar{\chi }\left[n\prime \right]\sim \bar{\chi }\left(t\right)$. Five different noise power values were simulated corresponding to SNR = 40, 20, 0, −20, and −40 dB, where SNR=10log{EN($\bar{y}\left[n\prime \right]$)/ EN($\bar{e}\left[n\prime \right]$)} and EN stands for the signal energy. Two different antialiasing low-pass filters (LPF1 and LPF2, see Figure 7b) were also applied using the command *filtfilt* to leave the resulting signal phases unaltered, with cut-off frequencies of $f_{c1}=1.22\;f_{2}$, $f_{c2}=1.15\;B$, respectively. Then, $\bar{y}\left[n\right]$ and $\bar{\chi }\left[n\right]$ were retrieved by sampling the resulting signals at $f_{ADC}$. A sketch of the implemented routine is depicted in Figure 7a, while Figure 7b shows the implemented signal processing for each of the explored algorithms.

As shown in Figure 7, two figures of merit were calculated for each scheme and SNR value in order to evaluate both the accuracy and the repeatability of distance estimation. In particular, the mean of the estimated d values was calculated over the $N_{trials}$, i.e., $d_{Corr}$, $d_{FMCW}$, and $d_{FMCW}^{Mod}$, respectively, for the correlation, standard, and modified FMCW schemes so as to evaluate the accuracy of each estimator. Additionally, a best fit line was calculated for each dataset so as to compute the overall standard deviations: $\sigma_{Corr}$, $\sigma_{FMCW}$, and $\sigma_{FMCW}^{Mod}$. By assuming an underlying Gaussian distribution, the standard deviations allow for assessing the repeatability of each measurement scheme with a 68.3% probability of confidence [59].

## 5. Results and Discussion

### 5.1. Numerical Results

**Figure 8:**
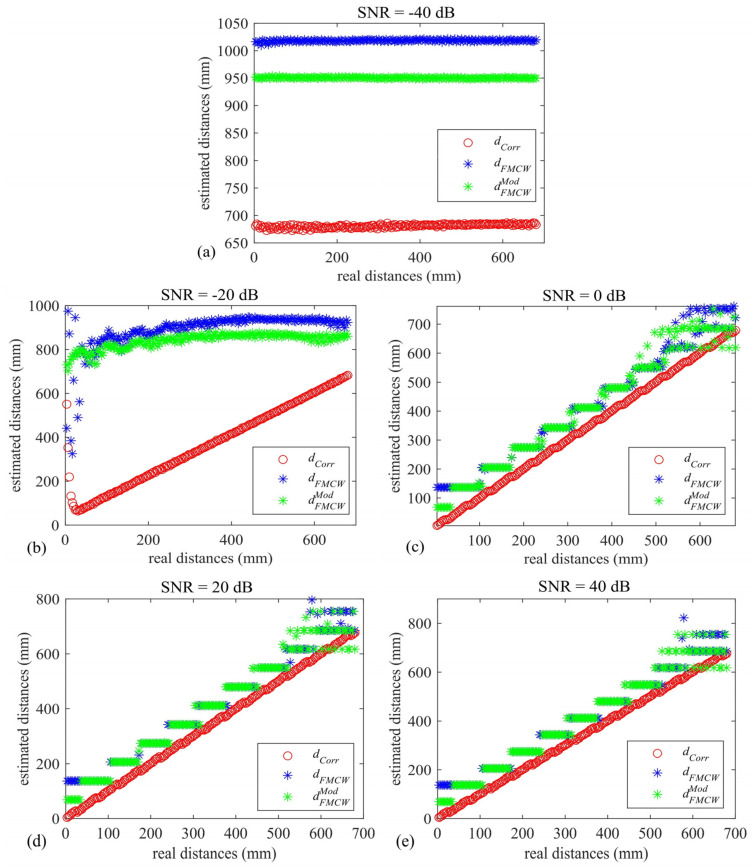
Estimated mean distance values vs. real distances for the correlation—(red circles), standard FMCW—(blue stars), and modified FMCW-based (green) star approaches at (**a**) −40 dB, (**b**) −20 dB, (**c**) 0 dB, (**d**) 20 dB, and (**e**) 40 dB.

Figure 8a–e depict the estimated $d_{Corr}$, $d_{FMCW}$, and $d_{FMCW}^{Mod}$ values with respect to the true simulated distances. It can be noticed from Figure 8a that all the methodologies fail to estimate the real distances at a very low SNR value, i.e., −40 dB. This is expected when choosing such narrowband transducers and relatively low $f_{ADC}$ values and signal time durations. At an SNR level equal in value to −20 dB (see Figure 8b), the correlation output tracks the real distances well, although misleading results are evident at very low distances, and those obtained using the standard and the modified FMCW approach outputs are completely out of range.

For increasing the values of SNR (see Figure 8c–e), all the techniques estimate the real distances very well, with the highest accuracy given by the correlation method, as expected. It can be seen that the modified FMCW approach estimates distances <50 mm slightly better with respect to what is achievable with the standard FMCW; a narrower distribution of the modified FMCW plot (green-starred) with respect to the standard FMCW is visible at distances < 100 mm. It is worth noting that the modified FMCW agrees well with the standard FMCW results across the whole inspected range, thus leaving the range estimation capability unaltered. In order to evaluate the repeatability of each of the employed schemes, Table 2 reports the value of the total standard deviation $\sigma_{Corr}$, $\sigma_{FMCW}$, and $\sigma_{FMCW}^{Mod}$ in millimeters for each of the SNR values, except for -40 dB. It can be seen that the repeatability of both the FMCW approaches tends toward that of the correlation approach as the SNR increases.

In order to better appreciate the potential of the modified FMCW scheme, it is worth simulating a measurement scheme that is not constrained by the digital nature of the hardware, a good example being the analog output of a PLL in the frequency-demodulation configuration for estimating the beat frequency after the FMCW. The PLL can be simply implemented onto the UGVs, bypassing the DAC of the Analog Discovery. Note that the PLL is commonly implemented via a voltage-controlled oscillator (VCO), an operational amplifier (op-amp), and a low-pass filter; thus, it can be set up on UGVs and drones without affecting the intended cost/weight ratio. A possible way to simulate such a circuit is to apply a three-point parabolic fit to the estimated TOF, i.e., to the result of Equation (13), so as to mimic the continuous-analog behavior of the PLL.

**Figure 9:**
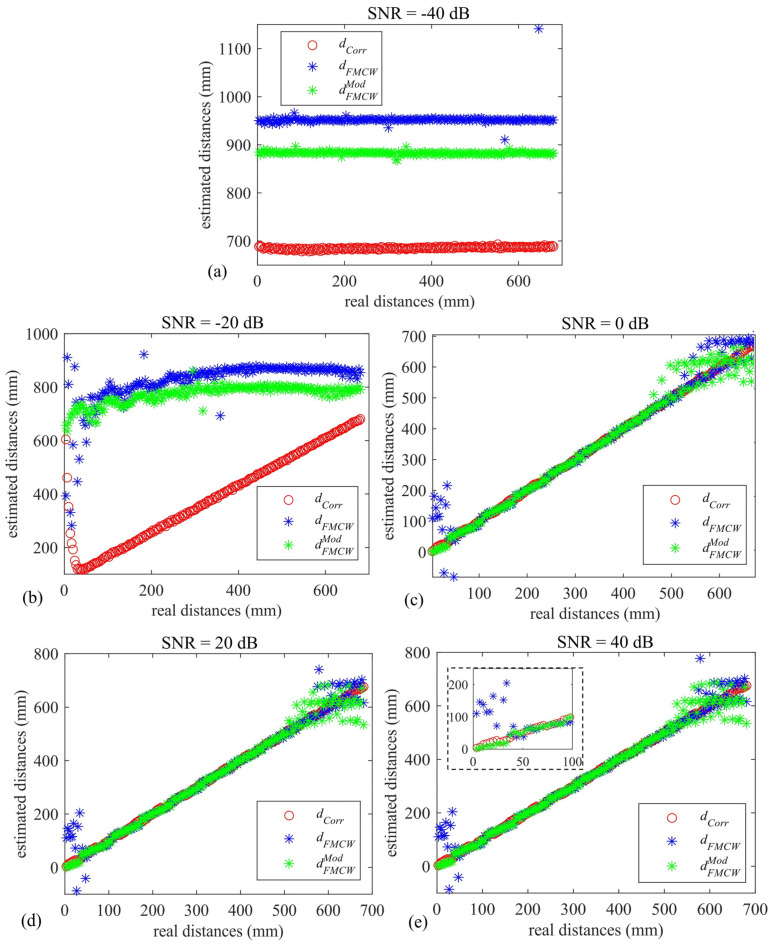
Estimated mean distance values vs. real distances for the correlation—(red circles), standard FMCW—(blue stars), and modified FMCW-based (green) star approaches at (**a**) −40 dB, (**b**) −20 dB, (**c**) 0 dB, (**d**) 20 dB, and (**e**) 40 dB, computed after a parabolic interpolation.

The results of this procedure for the correlation and the FMCW-based methods are shown in Figure 9. Even though the results at low SNR values (Figure 9a,b) are similar to the noninterpolated ones, a dramatic enhancement in accuracy is noticed for the remaining simulated values. In particular, it can be seen that the modified FMCW scheme outperforms the range estimation capabilities of the standard one, especially for distances below 50 mm, following the correlation output across the whole measurement range very well, starting from 0 dB of SNR. This is highlighted in Figure 9e, whereby a subplot containing a zoom of the three techniques within the range 0–100 mm is reported. Note that the two FMCW-based approaches tend to output the same results after the critical range distance, i.e., *d >* 68.6 mm. The computed $\sigma_{Corr}$, $\sigma_{FMCW}$, and $\sigma_{FMCW}^{Mod}$ after the interpolation are reported in Table 3. As expected, these are slightly lower than those achieved without the interpolation procedure, and they are consistent for the three evaluated techniques.

### 5.2. Experimental Results

**Figure 10:**
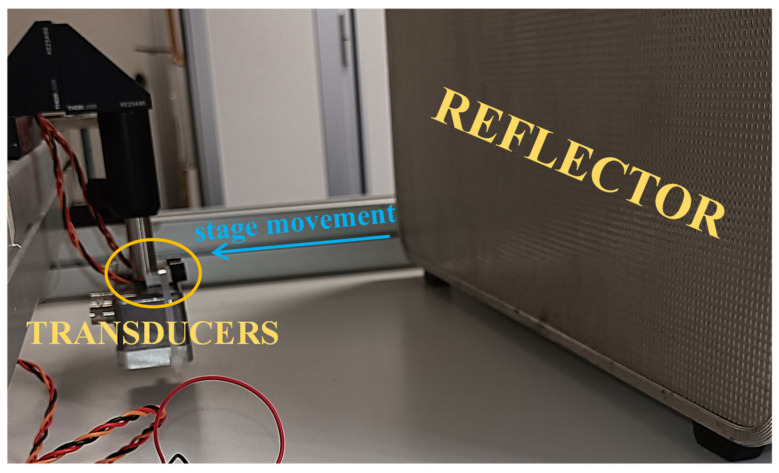
Photograph of the experimental setup.

Experimental measurements were performed to corroborate the promising findings of the numerical simulations using the same hardware and chirp characteristics described in the previous sections. Figure 10 shows a photograph of the experimental setup. The two transducers were arranged as pulse-echo, with their closest sides being 15 mm away from each other. The transducers were originally placed in contact with a large reflector (i.e., at 0 mm), this being a 4 mm-thick aluminum plate, and were then moved further away from it by means of an in-house motorized scanning stage. The overall distance range was 0–100 mm in steps of 2 mm; a total of 100 measurements were recorded at each increment of distance so as to gain robust statistics. The signal generation, standard and modified FMCWs implementation with the three-point parabolic fitting, and stage movement were managed using a Virtual Instrument (VI) developed in LabVIEW. The measurements were carried out at room temperature, i.e., 20 °C, where the speed of sound in air was 343 ms^−1^.

**Figure 11:**
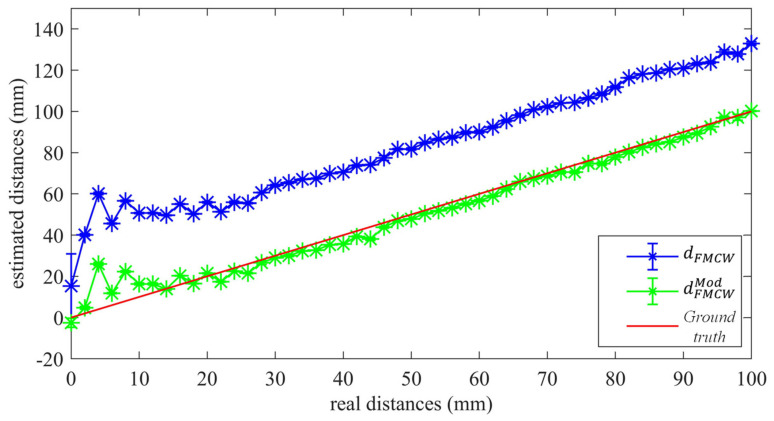
Experimental estimated distances and standard deviation values for both the standard (blue stars) and modified FMCW schemes, together with the ground truth (red line plot).

Figure 11 shows the estimated distances obtained after the parabolic fit for both the standard (blue stars) and modified FMCWs (green stars), together with the actual distance, as the straight line in red, i.e., the ground truth. Note that the standard deviation values of the 100 measurements collected at each considered distance are also reported in the form of error bars. It can be seen that the modified FMCW shows improved performance with respect to its standard counterpart; the modified scheme follows the ground truth well along the whole range of distances considered. It is worth noting that crosstalk between the two transducers plays a detrimental role at distances below 10 mm from the reflector: see the relatively-large estimation error of the two FMCWs approaches within the range of distances 6–10 mm. However, this problem can be tackled by using a suitable amount of sound-absorbing material placed in between the two transducers. Nevertheless, the modified FMCW approach outputs satisfactory results within the mentioned short range without employing any absorbing material, showing largely improved estimates for the 0 mm distance in both accuracy and repeatability with respect to the standard approach.

## 6. Conclusions and Future Work

An ultrasonic range and positioning system was investigated both numerically and experimentally to consider its possible application in unmanned ground vehicles. With this aim, the numerical simulations were taken into account for the features of an ultrasonic system, with a pair of low-frequency/narrowband ultrasonic transducers and an arbitrary waveform generator/digitizer, both of which are highly portable, light, and cheap.

A modified FMCW-based algorithm was introduced, and its capabilities for estimating distances in the range of 0–380 mm were numerically investigated, with particular emphasis on distances below 50 mm. This was carried out by comparing the proposed algorithm with both the widely-used correlation method and a standard FMCW counterpart.

The results showed that the proposed modified FMCW aids in estimating the distance to extremely near targets while leaving its estimation capabilities unaltered along a more extended inspection range. Finally, it must be noted that the proposed algorithm is of general validity for any value of transducer bandwidth, the choice of which will impose different limitations on the closest distances that can be estimated using the FMCW. A prototype of the proposed portable system implementing the modified FMCW scheme using a voltage-controlled oscillator for signal generation and a phase-locked loop to estimate the distance will be realized and set up on an unmanned ground vehicle in future work; its range estimation capabilities will be compared with other existing ranging and positioning schemes, such as MFSK. Its robustness regarding the Doppler effect will also be considered.

## Figures and Tables

**Table 1:** Classification of UAVs.

Category	Weight (kg)	Operating Altitude (m)	Nominal Endurance (h)	Payload (kg)
Micro	<2	<140	<1	<1
Mini	2–25	<1000	2–8	<10
Small	25–150	<1700	4–12	<50
Medium	150–600	<3300	8–20	<200
Large/Tactical	>600	>3300	>20	>200

**Table 2:** Computed standard deviations for each estimator and SNR value, considering the results shown in Figure 8.

		SNR (dB)				
		−40	−20	0	20	40
Total standard deviation (mm)	$\sigma_{Corr}$	-	9.47	9.63	9.62	9.62
	$\sigma_{FMCW}$	-	13.89	10.73	10.60	10.57
	$\sigma_{FMCW}^{Mod}$	-	12.50	10.59	10.54	10.55

**Table 3:** Computed standard deviations for each estimator and SNR value, considering the results shown in Figure 9.

		SNR (dB)				
		−40	−20	0	20	40
Total standard deviation (mm)	$\sigma_{Corr}$	-	9.40	9.62	9.61	9.61
	$\sigma_{FMCW}$	-	12.90	9.74	9.63	9.61
	$\sigma_{FMCW}^{Mod}$	-	11.54	9.61	9.55	9.56

## Data Availability

The data presented in this study are available on request from the corresponding author.
